# SMAD4–201 transcript as a putative biomarker in colorectal cancer

**DOI:** 10.1186/s12885-022-09186-z

**Published:** 2022-01-16

**Authors:** Tamara Babic, Sandra Dragicevic, Marko Miladinov, Zoran Krivokapic, Aleksandra Nikolic

**Affiliations:** 1 Institute of Molecular Genetics and Genetic Engineering, University of Belgrade, Vojvode Stepe 444a, 11042, Belgrade, Serbia; 2 Clinic for Digestive Surgery, Clinical Center of Serbia, Belgrade, Serbia; 3 Faculty of Medicine, University of Belgrade, Belgrade, Serbia; 4 Serbian Academy of Sciences and Arts, Belgrade, Serbia

**Keywords:** 5′-untranslated regions, Alternative transcripts, Colorectal cancer, SMAD4

## Abstract

**Background:**

Transcripts with alternative 5′-untranslated regions (UTRs) result from the activity of alternative promoters and they can determine gene expression by influencing its stability and translational efficiency, thus executing complex regulation of developmental, physiological and pathological processes. Transcriptional regulation of human *SMAD4*, a key tumor suppressor deregulated in most gastrointestinal cancers, entails four alternative promoters. These promoters and alternative transcripts they generate remain unexplored as contributors to the *SMAD4* deregulation in cancer. The aim of this study was to investigate the relative abundance of the transcript SMAD4–201 in colorectal cell lines and tissues in order to establish if its fluctuations may be associated with colorectal cancer (CRC).

**Methods:**

Relative abundance of SMAD4–201 in total SMAD4 mRNA was analyzed using quantitative PCR in a set of permanent human colon cell lines and tumor and corresponding healthy tissue samples from patients with CRC.

**Results:**

The relative abundance of SMAD4–201 in analyzed cell lines varied between 16 and 47%. A similar relative abundance of SMAD4–201 transcript was found in the majority of analyzed human tumor tissue samples, and it was averagely 20% lower in non-malignant in comparison to malignant tissue samples (*p* = 0.001). Transcript SMAD4–202 was not detectable in any of the analyzed samples, so the observed fluctuations in the composition of SMAD4 transcripts can be attributed to transcripts other than SMAD4–201 and SMAD4–202.

**Conclusion:**

The expression profile of SMAD4–201 in human tumor and non-tumor tissue samples may indicate the translational potential of this molecule in CRC, but further research is needed to clarify its usability as a potential biomarker for early diagnosis.

**Supplementary Information:**

The online version contains supplementary material available at 10.1186/s12885-022-09186-z.

## Background

SMAD family member 4 (SMAD4) is essential for the maintenance of tissue homeostasis and cell cycle regulation. This molecule is a key tumor suppressor in human gastrointestinal tissues and its expression was established as altered in various types of solid tumors [1]. The consequences of SMAD4 inactivation differ depending on tissue type [2]. Loss of SMAD4 is known to play a causal role in initiating gastrointestinal cancers, while in pancreatohepatobiliary cancers SMAD4 deficiency does not initiate tumorigenesis but acts as a promoter of a malignant process that was initiated by the other tumorigenic mechanisms.

Loss of tumor suppressor SMAD4 occurs in about 30% of colorectal cancer (CRC) cases [3]. In colorectal tumors, SMAD4-deficiency correlates with poor prognosis, metastases, resistance to 5-fluoruracil and disease recurrence [4–6]. Loss of heterozygosity results in a decreased level of the SMAD4 protein and it can have similar functional consequences as complete loss of SMAD4, consequently leading to intestinal tumorigenesis [7, 8]. Posttranscriptional regulation of the *SMAD4* gene can also be involved in colorectal carcinogenesis, through action and interaction of long and short non-coding RNAs [9, 10]. Alteration in the composition of transcripts with alternative 5′-untranslated regions (5′-UTRs) represents another potential dimension of *SMAD4* deregulation in CRC that remains unexplored. These untranslated regions of mRNAs can determine gene expression by influencing mRNA stability and translational efficiency [11]. Spatiotemporal expression of transcripts with alternative 5′-UTRs can control protein expression, thus executing complex regulation of developmental, physiological and pathological processes.

Transcripts with alternative 5′-UTRs are produced by alternative promoters present in the majority of human genes [12]. There is growing evidence on aberrant use of multiple promoters in malignant cell and also of the importance of the promoter choice and its precedence over the gene’s overall level of transcriptional activity [13–15]. According to the results of recent studies, the activity profile of alternative promoters may be an indicator of tumor characteristics and prognosis [15–17]. Alternative promoters are deregulated in malignant diseases across tissues and cancer types, and the promoter activity provides a more accurate prediction of cancer patient survival than gene expression itself [17].

The cellular composition of transcripts with alternative 5′-UTRs results from the combined activity of alternative promoters and other DNA regulatory regions, and their interaction with the cellular proteome. A tumor-specific profile of a set of transcripts with alternative 5′-UTRs was detected by exon arrays in CRC tissue in comparison to normal gut mucosa [16]. The same study found that these transcriptional alterations occur even in adenoma, which indicates that they represent an early event in the process of malignant transformation. Alternative 5′-UTRs harbor unexplored potential as a source of both biomarkers for early cancer diagnostics and targets for novel therapeutic strategies [18, 19].

A complex region spanning over 80 kb drives transcription of *SMAD4* gene and four segments with promoter activity have been identified in this region [20]. According to the RNA Annotation and Mapping of Promoters for the Analysis of Gene Expression (RAMPAGE) data from the project Encyclopedia of DNA Elements (ENCODE), the major contributor to the SMAD4 protein expression in most tissues is transcript SMAD4–201 (ENST00000342988.8) and it is ubiquitously expressed in different tissue types [21]. Beside SMAD4–201, transcript SMAD4–202 (ENST00000398417.6) encodes for full-length protein and, as such, is considered to contribute to SMAD4 protein pull in a cell [22]. Considering growing evidence on aberrant use of multiple promoters in malignant cell and the importance of SMAD4 for malignant transformation, the aim of this study was to investigate the relative abundance of the transcript SMAD4–201 in colorectal cell lines and tissues, and also in development and under stress, in order to establish if its fluctuations may be associated with CRC.

## Methods

### Transcripts data and primers

The sequences of human SMAD4 and mouse Smad4 transcripts that encode full protein (552 amino-acids in human and 551 amino-acids in mouse) were downloaded from the Ensembl database (www.ensembl.org). Forward primer of each primer pair for amplification of transcripts 201 and 202 was designed to ensure specific detection by annealing to the sequence present exclusively in the 5′-UTR of the targeted transcript (Table 1).

**Table 1:** Primers used for detection of human SMAD4 and mouse Smad4 transcripts

Transcript name (ID)	Forward and reverse primer sequence	Product length (bp)
Human SMAD4–201 (ENST00000342988.8)	For: 5′-GCCCAGGTTATCCTGAATAC-3′ Rev.: 5′-GCTCAGACAGGCATCATTAC-3’	187
Human SMAD4–202 (ENST00000398417.6)	For: 5’-GAGAAGGAAGGTTATCCTG-3′ Rev.: 5′-CGTAATAGACATATTGTCC-3’	158
Total human SMAD4	For: 5’-CACTACGAACGAGTTGTATCACC-3′ Rev.: 5′-CTTGATGGAGCATTACTCTGCAG-3’	71
Mouse Smad4–201 (ENSMUST00000025393.13)	For: 5’-GCCCAGGTCATCCTGCTCACC-3′ Rev.: 5′-GCTCAGACAGGCATCGTTAC-3’	188
Mouse Smad4–202 (ENSMUST00000114939.1)	For: 5’-CCTTGTGAAATGTGTTCTCATG-3′ Rev.: 5′-CCGACCAGCCACCTGAAGTCG-3’	429
Total mouse Smad4	For: 5’-CGACTTCAGGTGGCTGGTCGG-3′ Rev.: 5′-GGATTCACACAGACACTGTCAC-3’	149

**Fig. 1:**
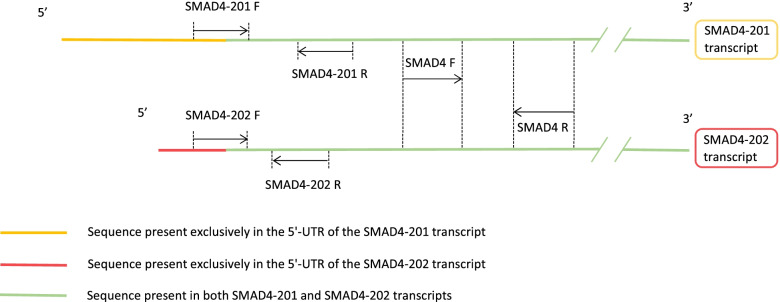
Schematic alignment of the two major SMAD4 transcripts and primers position. Human SMAD4 transcripts that encode full protein were aligned to distinguish between identical sequences in the coding part of the transcripts and 5’UTRs which discriminate them, in order to design appropriate primer pairs. Scheme refers to the mouse Smad4 transcripts, as human SMAD4 and mouse Smad4 transcripts are homologues and similar in length

Primer pairs for measurement of total SMAD4/Smad4 mRNA were designed to capture the sequence close to the 5’ end of the coding region, at the junction of the first two coding exons. Schematic alignment of the two major SMAD4 transcripts and primers position is shown in Fig. 1.

Glyceraldehyde-3-phosphate dehydrogenase was used as the internal housekeeping gene control in all experiments. Previously published primers were used for amplification of glyceraldehyde-3-phosphate dehydrogenase transcripts from human (NC_000012.12) and mouse (NC_000072.6) [23, 24].

### Cell lines

The following set of permanent human cell lines originating from colon tissue was used for the study: immortalized epithelial cells HCEC-1CT and six malignant cell lines: Caco-2, HCT116, HT29, DLD-1, SW480 and SW620. The study has also included cell lines from human fetal tissues, MRC-5 (lung fibroblasts) and HEK-293 (kidney epithelium). All cell lines were maintained at 37 °C and 5% CO_2_ in Dulbecco′s Modified Eagle′s - Medium (DMEM) supplemented with 10% fetal bovine serum, penicillin (10 U/mL), and streptomycin [25]. Cell line HCEC-1CT was subjected to treatments with 10 ng/mL of lipopolysaccharide (LPS) as an inducer of inflammation and 50 ng/mL of cigarette smoke extract (CSE) prepared from 1R3F standard research cigarettes as an inducer of oxidative stress. The treatments were performed in triplicates. The cells were seeded at a density of 300,000 in 35-mm cell culture dishes and 24 h after the seeding or the treatment the cells were lysed and total RNA was extracted using TRI Reagent Solution (Thermo Fisher Scientific) according to the manufacturer’s protocol.

### Tissue samples

The study has included 17 pairs of RNA samples extracted from tissue samples of 12 patients with primary CRC and 5 patients with metastatic CRC. Each pair of samples consisted of a resected rectal tumor tissue and an adjacent non-tumor tissue (intestinal mucosa for primary CRC and liver tissue for metastatic CRC). The samples were collected from patients who were diagnosed and surgically treated, and only patients who haven’t received preoperative chemoradiotherapy have been included in the study. The samples were collected at the Clinic for Digestive Surgery - First Surgical Clinic, Clinical Center of Serbia and ethical approval was obtained from the Ethical Committee of Clinical Center of Serbia, University of Belgrade. Informed consent was obtained from each subject of the study.

The study has also included RNA samples extracted from mouse liver from four different stages of development: 15 days old fetus, 20 days old fetus, 15 days old adult and 4 months old adult. All animal procedures were in compliance with the Directive 2010/63/EU on the protection of animals used for experimental and other scientific purposes, and the approval was obtained from the Ethical Committee for the Use of Laboratory Animals of the Institute for Biological Research “Sinisa Stankovic”, University of Belgrade.

### Quantitative real-time PCR (qRT-PCR)

The concentration and purity of all RNA samples were determined by UV absorption spectrophotometry at 260 nm/280 nm. Reverse transcription was performed using a High-Capacity cDNA Reverse Transcription kit (Applied Biosystems) according to the manufacturer’s protocol using 1 μg of RNA as a template. Expression of selected transcripts was measured in triplicate by quantitative real-time PCR (qRT-PCR) using Power SYBR Green PCR Master Mix (ThermoFisher Scientific). Melting curve analysis was performed for all reactions to ensure specificity of the products. Analysis was performed on 7500 Real-Time PCR System (Applied Biosystems), applying the 2^-dCt^ method for relative quantification. The difference between mRNA expression level of target genes and the GAPDH was expressed as 2^-dCt^ value where dCt was calculated according to the following formula: dCt = Ct _target gene_ – Ct _housekeeping gene_. The expression level of each analyzed transcript was calculated, normalized to endogenous control and compared with the total gene expression measured in the same sample.

### Statistical analysis

Statistical analysis was performed using Statistical Package for Social Sciences 20.0 (SPSS Inc., Chicago, Illinois, USA). To test the normality of data one sample Kolmogorov-Smirnov test was used. Differences between independent samples were analyzed by Kruskal-Wallis test, while differences between paired samples were analyzed by Related samples Wilcoxon signed-rank test. *P values* less than 0.05 were considered statistically significant.

## Results

The relative abundance of the transcript SMAD4–201 was analyzed in human malignant and non-malignant (adult and fetal) cell lines and tissue samples in order to explore its translational potential for colorectal cancer diagnostics. A homologous transcript in mouse (Smad4–201) was evaluated at different points during development for additional comparison of this transcript’s profiles between prenatal and postnatal tissues. The expression level of the transcript SMAD4–201 was also measured under stress in vitro to confirm that environmental factors don’t influence its fluctuations. In all experiments, the relative abundance of SMAD4–201 was calculated as a portion of total *SMAD4* expression, which was measured using primers that anneal to the beginning of the coding sequence.

**Fig. 2:**
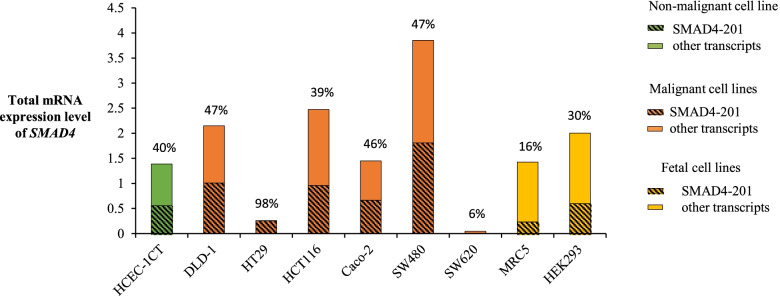
Total SMAD4 expression and relative abundance of SMAD4–201 transcript in colon and fetal cell lines. Data are presented as 2^-dCt^ values. Percentage values are representation of the relative abundance of SMAD4–201 transcript. HCEC-1CT - immortalized epithelial cells; Caco-2, HCT116, HT29, DLD-1, SW480, SW620 - malignant cell lines; MRC-5 – fetal lung fibroblasts; HEK-293 – fetal kidney epithelium

Detection of SMAD4–201 transcript was performed in a set of cell lines with different characteristics originating from colon tissue, two cell lines from human fetal tissue, and also in non-malignant colon cell line HCEC-1CT, which was additionally treated with LPS and CSE. Analyzed cell lines had a similar portion of SMAD4–201, between 16 and 47%, with the exception of cell lines HT-29 and SW620 (Fig. 2). While in HT-29 a very high portion of SMAD4–201 was observed (nearly 100%), in SW620 its abundance was much lower than in any other analyzed cell line (below 10%). In HT-29 and SW620 cell lines, total *SMAD4* expression was significantly decreased in comparison to the average values for human non-tumor tissue 25-fold and 145-fold, respectively. Analyzed human fetal cell lines had lower abundance of SMAD4–201 in comparison to analyzed colon cell lines - 19% in MRC-5 and 30% in HEK-293. In HCEC-1CT cells treated with LPS and CSE the portion of SMAD4–201 was similar to untreated control (*P* > 0.05).

**Fig. 3:**
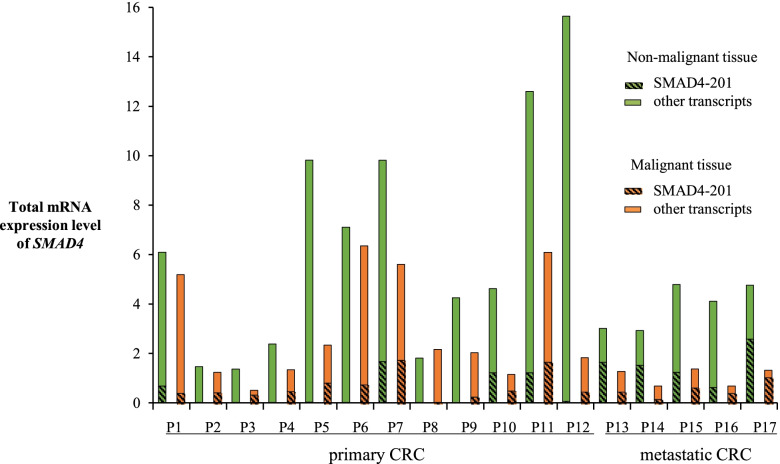
Total SMAD4 expression and relative abundance of SMAD4–201 transcript in patients with CRC. Malignant and non-malignant tissue samples have been analyzed for every patient. Data are presented as 2^-dCt^ values. P – Patients. Additional file 1 presents percentage value of the relative abundance of SMAD4–201 transcript for every patient

A set of human samples taken from patients with CRC was used to analyze the differences in SMAD4–201 expression levels between tumor and non-tumor tissue. Each tumor sample taken at biopsy during colonoscopy was paired with the sample of adjacent non-tumor tissue taken from the same patient. The abundance of SMAD4–201 transcript varied in both non-tumor tissue (between 0.07 and 26%) and tumor tissue (between 0.9 and 61%), while the overall fold change varied from 1 to 175 (Fig. 3). In all analyzed sample pairs but two, the portion of SMAD4–201 transcript was higher in tumor in comparison to non-tumor tissue, and the average increase was 20% (*p* = 0.001).

**Fig. 4:**
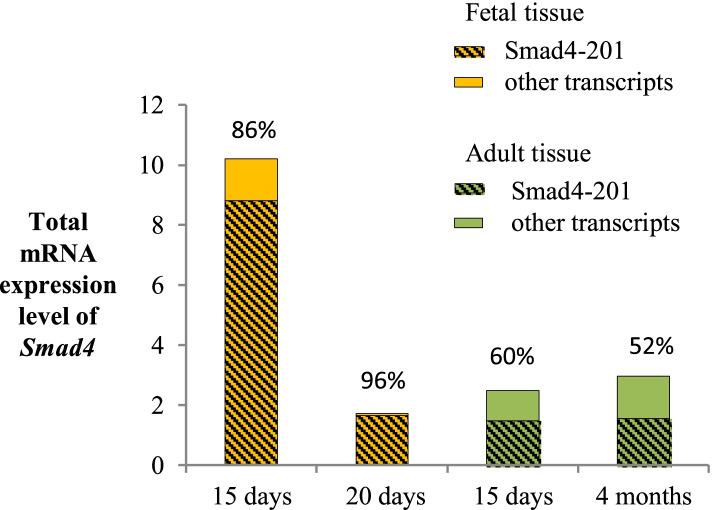
Total Smad4 expression and relative abundance of Smad4–201 transcript in mouse. Mouse tissues from different points during development have been analyzed. Data are presented as 2^-dCt^ values. Percentage values are representation of the relative abundance of Smad4–201 transcript

Mouse transcript Smad4–201 was detected at two prenatal stages (15 and 20 days) and two adult stages (15 days and 4 months). Its contribution to total Smad4 mRNA decreases after birth, but it remains dominant (over 50%) in adult tissue (Fig. 4).

The abundance of the only other fully coding SMAD4 transcript (SMAD4–202 in human and Smad4–202 in mouse) was also analyzed in all samples. In mouse, this transcript was detected in trace amounts, while in human cell lines and samples it was undetectable.

## Discussion

The purpose of this study was to investigate the transcript SMAD4–201 as a potential biomarker for CRC. The relative abundances of this transcript and the transcript SMAD4–202 that also codes for full protein were analyzed in permanent human cell lines, and in a set of tumor and corresponding healthy tissue samples from patients with CRC. The analysis of homologous transcripts was also profiled in human fetal cell lines and mouse tissue in order to establish their dynamics during the prenatal and postnatal periods. The portion of SMAD4 transcripts in total SMAD4 mRNA was measured as a ratio of transcript expression and total SMAD4 expression. The primers binding to the beginning of the SMAD4 coding sequence were employed to measure total SMAD4 mRNA in order to achieve detection of all SMAD4 transcripts even in those samples affected by tumor driver mutations, which can occur in either downstream parts of N-terminal coding part of the gene or anywhere within the C-terminal coding region [26].

The relative abundance of SMAD4–201 has varied greatly across analyzed samples. In some non-tumor colorectal tissue samples this transcript was barely detectable (below 0.01%), while in fetal mouse liver it constituted almost total Smad4 mRNA (over 95%). Considering that the transcript SMAD4–202 was undetectable, fluctuations in the composition of SMAD4 transcripts in analyzed samples have to be attributed to change in levels of other, yet unidentified transcripts. The future profiling of SMAD4 transcripts with alternative 5′-UTRs should be performed using techniques for sequencing of RNA 5’ ends, such as CAGE-seq, RAMPAGE or STRIPE-seq to ensure that the entire pull of SMAD4 5′-UTRs is qualitatively and quantitatively profiled [27].

The analyzed colon cell lines had a similar portion of SMAD4–201, between 38 and 47%, including the non-malignant cell line HCEC-1CT. Considering that this cell line is immortalized by telomerase reverse transcriptase and cyclin-dependent kinase 4, although it is not malignant, it is characterized by replicative immortality that is an important hallmark of cancer, and therefore its transcriptome does not fully reflect that of the healthy tissue. Of the analyzed malignant cell lines, DLD-1 belongs to the consensus molecular subtype of colorectal cancer CMS1, while HCT116, Caco2, SW480 and SW620 belong to the CMS4 group, and no significant difference was observed in SMAD4–201 abundance between these two CMS groups [28, 29]. Cell lines MRC-5 and HEK-293 that originate from fetal tissue had a slightly lower abundance of SMAD4–201 transcript. Cell lines HT-29 and SW620 contained very high (almost 100%) and very low portions of SMAD4–201 (below 10%), respectively. The extremely low levels of total *SMAD4* expression obtained for these two cell lines could explain the obtained portions of SMAD4–201 that differed significantly from the other analyzed cell lines.

The analyzed colorectal tissue samples varied in content of the SMAD4–201 transcript and its abundance was increased for an average of 20% in malignant in comparison to non-malignant tissue (*p* = 0.001). The abundance of SMAD4–201 in non-malignant tissue was extremely low in most samples (below 27%), and in all malignant samples but two this value was increased between 1 and 175 times. The results obtained for transcript SMAD4–201 in human tumor and non-tumor tissue samples may indicate the translational potential of this molecule as a putative CRC biomarker. However, additional research is needed to refine and clarify SMAD4–201 potential as a biomarker due to the small number of clinical samples in our study. A larger-scale study would further elucidate the applicability of this candidate biomarker for screening and follow up purposes. The other transcripts contributing to the total SMAD4 expression are dominant in non-tumor tissue and they should be further characterized, since their decrease and/or loss seems to be associated with carcinogenesis. The great variability observed among the samples could be explained by tumor heterogeneity, but analysis of larger series of samples is still necessary in order to evaluate the biomarker potential of SMAD4–201. The relative abundance of SMAD4–201 detected in the analyzed samples was quite lower than expected based on ENCODE RAMPAGE data. The relative abundance value was below 30% in non-malignant tissue, and in two thirds of samples it was below 10%. Since the available data for comparison from the ENCODE project are obtained on colon mucosa and data obtained in that project for other tissues indicate high variability across tissue types, the lower abundance of SMAD4–201 transcript obtained for tissue samples may be due to the fact that the tumor samples for this study came almost exclusively from rectal tissue. According to ENCODE RAMPAGE data, there is a slight difference in the distribution of SMAD4–201 transcript levels between the sigmoid and transverse colon (0.11–0.29 vs. 0.28–0.42), which represent the same tissue type from different locations within the organ [21]. Rectal tissue is expected to be more similar to the sigmoid tissue than to the distal parts of the gut epithelium, including transversal colon. Considering the differences obtained for transcripts profiles in this study and also by the ENCODE project, future studies of human samples should include samples from different tumor locations.

The early translational potential of SMAD4–201 transcripts might be confirmed by demonstrating that its expression levels are unaffected by extracellular stressors in HCEC-1CT cell line. The oxidative stress was induced using CSE prepared from 1R3F standard research cigarettes at the concentration equivalent to the upper limit of the range of nicotine amount in the blood of smokers [30]. The inflammation was induced using LPS at the concentration sufficient to induce alterations in cellular signaling and metabolism [31]. Under both treatments, the relative abundance of SMAD4–201 remained the same.

Mouse was used as a suitable model system to investigate the relation between SMAD4 transcripts dynamics of developing and adult tissue, since the sequence similarity between human and mouse SMAD4 is over 98%. Although transcripts 201 of human and mouse are homologues and similar in length, as are transcripts 202, sequence alignment of human-mouse transcript pairs demonstrated low similarity between their 5′-UTRs. In spite of that, human and mouse transcripts 201 and 202 most likely exert similar functions and their similar patterns of expression were expected in these two organisms. Transcript 201 was found to remain dominant (over 50% of all Smad4 transcripts) in the mouse tissue during both prenatal and postnatal periods. This result is expected, since transcript 201 is encoded by a typical promoter, and such promoters are mostly ubiquitously expressed [32]. The transcript 202 was present at very low amounts in all analyzed mouse tissue samples (below 0.03%) and it doesn’t contribute significantly to total Smad4 mRNA. The total SMAD4 expression was similar between analyzed fetal human cell lines and fetal mouse tissue. However, the relative abundance of SMAD4–201 was quite different between these sample types and it was much higher in mouse tissue than in analyzed cell lines (Figs. 2 and 4). The results obtained for human fetal cell lines are in line with the ENCODE RAMPAGE data for prenatal human tissue, but such data are not available for mouse. Considering that the analyzed time points in mouse development are closer to birth than the time points in human development represented by the analyzed fetal cell lines, it is possible that SMAD4–201 fluctuates dynamically during the prenatal period, and other time points in human and mouse development should also be analyzed. It should also be noted that transcriptional and translational turnover in mouse is quite higher than in human [33].

The observed dynamics in the content of SMAD4–201 in human and mouse adult and developmental tissue is in line with the concept that alternative transcription initiation represents a mechanism normally occurring during prenatal development, while in postnatal period it is most commonly associated with pathology [34]. An alternative promoter usage appears to be yet another characteristic of malignant cell that resembles the developing cell and the phenomenon of aberrant alternative promoter usage has been associated with cancer [35, 36]. More recent studies indicate mutations of alternative promoters as a mechanism leading to aberrant usage of one promoter over the other [37, 38]. Functionally relevant mutations in alternative *SMAD4* promoters were shown to be quite rare, so in the case of this tumor suppressor other mechanisms and interactions with the cellular proteotranscriptome may be more relevant [39–42]. The cellular content of transcripts with alternative 5′-UTRs results from a variety of factors and the exact mechanisms behind the process of aberrant alternative transcription initiation in cancer remains to be elucidated.

## Conclusions

Loss of tumor suppressor SMAD4, its decreased level, and posttranscriptional regulation are all known mechanisms involved in colorectal carcinogenesis. However, the use of *SMAD4* alternative promoters and transcripts they generate in the malignant cell remain unexplored as contributors to the SMAD4 deregulation in cancer. Transcript SMAD4–201, a transcript that encodes for full-length SMAD4 protein, has a solid potential for further investigation as a biomarker for early diagnosis of CRC, since its relative abundance was increased for an average of 20% in malignant in comparison to non-malignant tissue. This research helps to enlighten the role of the SMAD4–201 transcript in colorectal cancer and its translational potential as a predictive and prognostic biomarker. Further research, including a larger number of clinical samples originating from different locations of the gut, should provide a more complete perspective on the potential of this molecule as a candidate biomarker. This line of research could open a window towards a novel therapeutic strategy in cancer, as the modulation of alternative transcription initiation and mRNA untranslated regions provides an opportunity to target molecules that are considered undruggable in malignant diseases [19, 43].

## Supplementary Information

**Additional file 1.** Percentage value of the relative abundance of SMAD4–201 transcript for every patient in malignant and non-malignant tissue

## Data Availability

The datasets used and/or analyzed during the current study are available from the corresponding author on reasonable request.

## Abbreviations

UTR: Untranslated region
CRC: Colorectal cancer
RAMPAGE: RNA Annotation and Mapping of Promoters for the Analysis of Gene Expression
ENCODE: Encyclopedia of DNA Elements
DMEM: Dulbecco′s Modified Eagle′s – Medium
LPS: lipopolysaccharide
CSE: Cigarette smoke extract
qRT-PCR: Quantitative real-time PCR
