# Supplementary material for: SMAD4–201 transcript as a putative biomarker in colorectal cancer
Source: BMC Cancer. 2022 Jan 16;22:72. doi: 10.1186/s12885-022-09186-z (PMC8762975; doi:10.1186/s12885-022-09186-z)
Supplement: Supplementary file 1 — Additional file 1. Percentage value of the relative abundance of SMAD4–201 transcript for every patient in malignant and non-malignant tissue [file 12885_2022_9186_MOESM1_ESM.docx]

|  | Patient | Non-malignant tissue (%) | Malignant tissue (%) |
| --- | --- | --- | --- |
| Relative abundance of SMAD4-201 transcript | P1 | 11 | 8 |
|  | P2 | 1 | 33 |
|  | P3 | 1 | 61 |
|  | P4 | 1 | 34 |
|  | P5 | 1 | 34 |
|  | P6 | 1 | 11 |
|  | P7 | 17 | 31 |
|  | P8 | 1 | 1 |
|  | P9 | 1 | 12 |
|  | P10 | 26 | 42 |
|  | P11 | 10 | 28 |
|  | P12 | 1 | 24 |
|  | P13 | 54 | 34 |
|  | P14 | 52 | 20 |
|  | P15 | 26 | 44 |
|  | P16 | 15 | 56 |
|  | P17 | 54 | 75 |

**Additional file 1. Percentage value of the relative abundance of SMAD4-201 transcript for every patient in malignant and non-malignant tissue.**
